# Silica and graphene mediate arsenic detection in mature rice grain by a newly patterned current–volt aptasensor

**DOI:** 10.1038/s41598-021-94145-0

**Published:** 2021-07-19

**Authors:** M. N. A. Uda, Subash C. B. Gopinath, Uda Hashim, N. H. Halim, N. A. Parmin, M. N. Afnan Uda, Tijjani Adam, Periasamy Anbu

**Affiliations:** 1 Institute of Nano Electronic Engineering, Universiti Malaysia Perlis, 01000 Kangar, Perlis Malaysia; 2 Faculty of Chemical Engineering Technology, Universiti Malaysia Perlis, 02600 Arau, Perlis Malaysia; 3 Faculty of Electronics Engineering Technology, Universiti Malaysia Perlis, 02600 Arau, Perlis Malaysia; 4 Department of Biological Engineering, College of Engineering, Inha University, Incheon, Republic of Korea

**Keywords:** Biochemistry, Nanoscience and technology

## Abstract

Arsenic is a major global threat to the ecosystem. Here we describe a highly accurate sensing platform using silica nanoparticles/graphene at the surface of aluminum interdigitated electrodes (Al IDE), able to detect trace amounts of arsenic(III) in rice grain samples. The morphology and electrical properties of fabricated Al IDEs were characterized and standardized using AFM, and SEM with EDX analyses. Micrometer scale Al IDEs were fabricated with silicon, aluminum, and oxygen as primary elements. Validation of the bare Al IDE with electrolyte fouling was performed at different pH levels. The sensing surface was stable with no electrolyte fouling at pH 7. Each chemical modification step was monitored with current–volt measurement. The surface chemical bonds were characterized by fourier transform infrared spectroscopy (FTIR) and revealed different peaks when interacting with arsenic (1600–1000 cm^−1^). Both silica nanoparticles and graphene presented a sensitive limit of detection as measured by slope calibration curves at 0.0000001 pg/ml, respectively. Further, linear regression was established using ΔI (A) = 3.86 E^−09^ log (Arsenic concentration) [g/ml] + 8.67 E^−08^ [A] for silica nanoparticles, whereas for graphene Y = 3.73 E^−09^ (Arsenic concentration) [g/ml] + 8.52 E^−08^ on the linear range of 0.0000001 pg/ml to 0.01 pg/ml. The R^2^ for silica (0.96) and that of graphene (0.94) was close to the maximum (1). Modification with silica nanoparticles was highly stable. The potential use of silica nanoparticles in the detection of arsenic in rice grain extract can be attributed to their size and stability.

## Introduction

Arsenic is an extremely toxic substance that can be found in both natural and anthropogenic sources around the world^1^. It is a metalloid that represents a major worldwide challenge to biodiversity and community health^2^. Arsenic is naturally found in both organic like arsenate [As(V)] and inorganic forms like arsenite [As(III)]^3^. Long-term exposure to this metalloid causes health problems, such as skin damage, circulatory system problems, and cancer by enhancing tumor growth^4^. Even low doses of this metalloid have been associated with various medical complications provoked by acute poisoning, known as “arsenicosis”^5^.

Due to its widespread availability and abundance across the globe, several studies have reported high levels of arsenic that exceeded permissible limits in natural sources (e.g., drinking or irrigation water), in a variety of crops (e.g., cereals, fruits, and vegetables), and animal protein sources (e.g., meat, fish, and poultry)^5–8^. The World Health Organization specifies that the maximum concentration limit of arsenic in drinking water is 0.01 mg/l (0.13 M)^9,10^. The Food and Agriculture Organization set the maximum concentration limit of arsenic in irrigation water at 0.10 mg/l^11^.

Numerous methods for screening and detecting arsenic at low doses have been applied, particularly atomic absorption spectrometry^12^, atomic fluorescence spectrometry^13^, and inductively coupled plasma^14^. However, these methods have some drawbacks: their maintenance is expensive, they require well-trained professionals for the analysis of the sample, and the in-situ application is extremely difficult, requiring the translocation of the sample to the laboratory for further analysis. As a result, the need for alternative, time- and cost-effective solutions that would permit real-time in situ arsenic detection, is imperative.

Biosensors are a potentially valuable technique for the identification of metalloids due to their specificity and sensitivity^15^. They recognize biomarkers, such as enzymes, antibodies, and aptamers, functioning as bio-components or chemical receptors^15–18^. Aptamers have several benefits as biomarkers over other molecules: they are small, easy to modify, inexpensive to manufacture, and can be generated to recognize a wide range of target molecules^19^. The characteristics of aptamers are similar to the antibody-binding mechanism, being highly specific to the target^20^.

Aptamers are smaller sized nucleic acid ligands with single-stranded-deoxyribonucleic acid (ssDNA) or -ribonucleic acid (RNA) or peptide molecule^20^. Their binding affinity is target-specific even at low femto-molecular scales for a variety of targets from smaller molecule to the whole cell^21^. The specific binding is due to the secondary structure of aptamer with stem and loop arrangements. Usually, the target molecule binds to the loop region and the stem areas provide stability to the aptamer. Overall, three common binding phenomena have been proposed, they are aptamer-induced target binding, target-induced aptamer binding or co-induced binding^22^. Apart from these properties, aptamers have shown considerable promises in sensing applications due to their ease in synthesis and modification with the desired functional groups and linkers^23^. Additionally, aptamers are excellent examples of functional biological molecules selected in vitro owing to their adaptability^24^. These benefits converted aptamers to a key player in various fields of research and applications such as in aptasensors^25^.

Furthermore, electrochemical techniques are used as sensing systems for quantification analyses. They present several advantages since they are low-cost, easy to operate, and have the potential of miniaturization; these characteristics make them useful in generalized transduction analysis and biosensor applications^26^. The combination of aptamers with electrochemical techniques is widely known as an electrochemical aptasensor^27^. However, the electrochemical aptasensor strategy without signal amplification often suffers from relatively poor detection limits^28^. Thus, the improvement of various parameters and the enhanced sensitivity of detection through engineered nanostructures have recently gained attention^29^. The nanostructures lead to an increase in the surface area of device by efficiently reorganizing the target during the measurement^30^.

Due to the nanoscale in dimension, the development of high-performance nanostructures such as silica nanoparticles and graphene was widely performed. Silica nanoparticles and graphene nanostructures were commonly recorded as vital nanomaterials created using various methods by top-down and bottom-up processes^30,31^. Specifically, silica nanoparticles have homogeneous pore size, hydrophilic and excellent surface properties. Moreover, silica has capability to increase the electrochemical active centers, improve electron transport and stimulates electrolyte penetration that fulfill the needs of energy storage devices of the future generation^32,33^ whereas graphene comprising sp2 carbon atomic orbital hybridization, it has a large specific surface area, great electron transport capacity and great mechanical strength, which are crucial for building the biosensors^34^. With biosensors silica and graphene have been used widely due their surface groups to be amenable for the functionalization and provides higher non-fouling. Further, silica acts as a potential layer and graphene is for the surface enhancement material.

Researchers have described different electrochemical aptasensors for arsenic detection by employing electrochemical impedance spectroscopy (EIS), cyclic voltammetry (CV), differential pulse voltammetry (DPV), current–voltage (IV) and other electrochemical signals with integrating nanomaterials (Table 1). As an example, Baghbaderani and Noorbakhsh have developed a device based on glassy carbon electrode as conductive platform and integrating with chitosan-nafion (Chit-Naf) nanomaterial to detect As(III)^35^. From this experiment, the researchers achieved to demonstrate that when a device was modified with nanomaterials, its capability is enhancing the kinetics of electron transport compared to the condition without modification, which possess a LOD of 0.74 nM. This system has the good features of reproducibility, selectivity, mediation-free interface renewability and beneficial for rapid As (III) detection in environmental samples.

**Table 1:** Currently available electrochemical aptasensors for arsenic analysis.

Electroanalytical method	Fabrication hierarchy scheme	Integrating strategy with nanomaterials	Blocking reagent	Buffer rinsed solution	Linear detection range	LOD	References
EIS	GC/Chit-Naf/GLA/DNAcap/BSA/Apt/GLA/CNTCOOH-BSA electrode	Chitosan-Nafion (Chit-Naf) with glassy carbon electrode (GCE)	Bovine serum albumin (CNT-BSA)	Phosphate-potassium chloride-sodium chloride buffer and distilled water	1.0–500 nM	0.74 nM	^35^
EIS	GC/Chit-Naf/GLA/DNAcap/BSA/Apt	Chitosan-Nafion (Chit-Naf) with GCE	CNT-BSA	Phosphate-potassium chloride-sodium chloride buffer and distilled water	0.15–100 nM	7.4 × 10^–2^ nM	^35^
DPV	GCE/CNPs/AuNPs/Aptamer/MCH electrode	Carbon nanoparticle (CNPs) and gold nanoparticles (AuNPs) with GCE	6-Mercaptohexanol (MCH)	Deionised water and dried with argon	0.5 ppb to 100 ppb	0.092 ppb	^36^
DPV	SPCE/Chit/AuNPs/Apt-Thiol/MCH electrode	AuNPs	MCH	Ultrapure water and drying with nitrogen	0.2 nM to 100 nM	0.15 nM	^37^
EIS	GCE/AuNPs/3D-rGO/Apt	3D-rGO/AuNPs with GCE	–	Ultrapure Water	3.8 × 10^–7^‒3.0 × 10^–4^ ng/ml	1.4 × 10^–7^ ng/ml	^38^
Cyclic Voltammetry (CV)	FET/CPPy-CFMNSs/Apt/Arsenic	Carboxylic polypyrrole (CPPy)-coated flower-like MoS2 nanospheres (CFMNSs)	–	Distilled water	1 pM to 10 nM	1.0 × 10^–3^ nM	^39^
DPV	SPE/AuNPs/Ars/GO-MB	Graphene oxide (GO) and methylene blue	Tris (2-carhoxyethyl) phosphine	Phosphate buffer saline (PBS)	0.01 and 0.1 mg/l	2 × 10–4 mg/l	^40^
I-V	AIIDE/ CDI/Streptavidin/Ethanolamine/Biotinylated aptamer/As	Silica Nanoparticles (SiNPs)/Graphene	Ethanolamine	PBS and distilled water	0.0000001 to 0.01 pg/ml	0.0000001 pg/ml	This work

Presently in this study, we developed an electrochemical aptasensor with a modified surface using silica nanoparticles or graphene on aluminum interdigitated electrodes (Al IDE) to detect arsenic. Al IDE is one of the most popular sensors that was widely used since 1960s due to its ability to be changed to improve detection efficiency^41^. Hereby, we provide a novel design for Al IDE, which functions at low voltage, it is cheap, portable, and fulfills the requirements of high-performance sensing. The chemical, physical, and electrical characteristics of the Al IDEs were studied using mature rice grain samples for the detection of arsenic.

## Experimental methods

### Materials

Phosphate buffer solution (PBS; 100 mM, pH 7.4), 1,1-carbonyldiimidazole (CDI), streptavidin, sodium (meta)arsenite (≥ 90%), ethanolamine (≥ 98.0%) were bought from Merck KGaA, USA. The aptamer sequence was generated by the in vitro selection and amplification technology SELEX. More specifically, a random DNA library was used to prepare the aptamers with modification at the 5′ end of 124-mer [5′-GGTAATACGACTCACTATAGGGAGATACCAGCTTATTCAATTTTACAGAACAACCAACGTCGCTCCGGGTACTTCTTCATCGAGATAGTAAGTGCAATCTAAAAAAAAAAA AAAAAAAAAAAAA-3′] (Apical Scientific Sdn. Bhd.; Kuala Lumpur, Malaysia)^42^. Biotin-dT20 was used for coupling chemistry at a concentration of 25 nmol DNA oligo (Integrated DNA Technologies, USA). The pH buffer solutions were used for chemical characterization through pH measurements (HANNA Instruments, Malaysia) and the pH values were measured with a commercial pH meter (Mettler Toledo F20; Mettler-Toledo (M) Sdn Bhd, Malaysia). All experiments have been done with a plant material complied to relevant institutional guidelines and authors grown under green house to analyse the rice grains with prior permission. The rice grain used is the identified commercial variety and permitted for the experimental purposes.

### Synthesis and characterization of silica nanoparticles and graphene

Rice straw was used as the primary raw material for the extraction of silica nanoparticles and graphene. It was collected from Perlis's rice mill in Simpang Empat Perlis, Malaysia. After drying, the cleaned paddy straw was subjected to a burning procedure, which resulted in the formation of ash. For the extraction for silica nanoparticles, used ~ 20 g of rice straw ash suspended in 400 ml of 2.5 M sodium hydroxide. At 100 °C for 4 h, ash and chemical combination was mixed. After several minutes, the mixture was sieved using Whatman No. 5 filter paper (47 mm). The residues were added in a titration process with 2 M sulphuric acid until equilibrium (neutral pH) was reached, and stirring was sustained for 18 h. After creating two distinct gel layers in the solution, stirring was discontinued. Centrifugation (6000 × *g* for 5 min) of the collected layers was resulted in the collection of the pellet. Then washed three times, once with ethanol and twice with distilled water. Following that, a thermal oven was used to dry the purified pellet for 30 min at 80 °C. Then, using a mortar and pestle, the pellet was finely ground to a powder.

For graphene extraction, ~ 3 g rice straw ash was mixed with 12 g potassium hydroxide and then ground using a mortar and pestle. The mixture of rice straw ash and KOH was compacted in a porcelain crucible. For two hours, the mixed sample was placed in a muffle furnace and subjected to annealing at 700 °C. Following that, the mixture was stirred for 6 h in 100 ml of clean water. Then, the sample was washed with distilled water to remove the excess KOH using a centrifugation (6000 × *g*, 5 min). Finally, the material was prepared for further evaluation after being dried in an electrical microwave at 150 °C for 24 h.

The morphological properties of silica nanoparticles and graphene were investigated using a Field Emission Scanning Electron Microscope (FE-SEM) from Hitachi, S-4300 SE, Japan, and a Field Emission Transmission Electron Microscope (FE-TEM) from Hitachi, S-4300 SE, Japan (Model: JEM 2100F). A drop of powder composed of silica nanoparticles and graphene was placed on a specified grid and energized at a rate of 10 Ampere. The particles were completely recognizable using the image processing software. Further, X-ray diffraction (XRD) was used to characterize the crystalline structure of generated silica nanoparticles and graphene. using a DMAX-2500 (Rigaku, Japan). A nickel filter and a Cu Ka (1.54059 A) radiation source were used to prepare the operational setting. Scanning was performed at a rate of 0.5/s with a diffraction angle range from 10° to 90°. At a voltage of 45 kV and a filament current of 40 mA, the operating system was observed.

### Design of aluminum interdigitated electrodes (Al IDE)

Al IDEs were designed on AutoCAD software VERSION 2018 (Malaysia) specifying the following parameters: the length, width, and shape of the electrode fingers, and size of the mask layout. All the parameters were designed according to a previous fabrication process of commercial Al IDEs (Silterra Sdn. Bhd., Malaysia). All the reagents were in analytical grade and used as instructed by the suppliers.

### Physical characterization of Al IDEs

#### Scanning electron microscope (SEM) and energy dispersive X-ray analysis (EDX)

To confirm the physical properties of the finger electrodes on the active surface of the fabricated Al IDEs, we applied SEM. EDX was used to confirm the depositions of silica substrate along with the aluminum. The morphological images were readily observed using the image analysis processing software (Elphy Quantum)^43^ and were used to determine the elemental composition of the Al IDEs in their entirety.

#### Atomic force microscopy (AFM)

The morphology of the surface was evaluated by AFM to reveal the Al IDE topography. Images were taken using a scanning probe microscope (Nano III, CA, USA). The scanning probe applied nitrite tips on contact mode at a frequency of 300 kHz. The pictures of the height, width, and cross-section were designed and calibrated from the topographic images.

### Electrical characterization of Al IDEs

#### Current–voltage (I–V) measurements

Five bare Al IDE devices were used for electrical characterization. This analysis was applied to test the Al IDEs´ performance without any shortage. Briefly, the Al IDEs were covered with distilled water (2 µl) and the consequent changes in current were observed and recorded. The measurements of I–V were performed using a picoammeter/voltage source in the range of 0–1 V. The results were transferred to Microsoft Excel with the Kick Start software.

#### Characterization of the Al IDEs by electrolyte scouting

About 2 µl of seven different solutions (pH 1, 3, 5, 7, 8, 11, and 13) were used to cover the finger gaps of the sensing surfaces of the Al IDEs. The changes in current were tracked to determine the effect of electrolytes at various pH levels on the sensor's surface. The I–V measurements at different pH levels were recorded using the same voltage source as previously mentioned.

#### Surface functionalization of aptamer-based Al IDE for the detection of As(III)

For the surface functionalization of the Al IDEs with a 5 µm finger-gapped channel, we incubated the sensing surface with 1,1-carbonyldiimidazole (CDI) for 1 h. This compound s coupled with the amidation of the carboxylic group^44^. Then, they were washed with PBS and consecutively incubated in streptavidin for 1 h. Streptavidin is widely used for the induction of a non-covalent interaction with biotin^45^. The remaining reactive groups had to be masked to avoid the covalent binding of the analyte to the reactive surface. To achieve this, ethanolamine was used as a blocking agent for 30 min, after washing the surface area of the finger gap with PBS. Next, the biotinylated aptamer was put on the surface of the AI IDE. In this experiment, 5′ biotin-labeled aptamer specific for the detection of As (III) was immobilized by cross-linking it with streptavidin. Then, we measured I–V measurement of the biotinylated aptamer after 5 min of interaction with arsenic. All the incubation processes for surface functionalization were carried out at room temperature.

### Limit of detection (LOD), analysis of spikes, and stability: high-performance

The high-performance aptasensor was confirmed by measurements of electrical conductivity using a picoammeter. To establish the sensitivity of the aptamer against arsenic, the analysis was performed with serial dilutions of sodium (meta)arsenite from 0.0000001 pg/ml to 0.01 pg/ml. The I–V values were calculated after the interaction of the immobilized aptamer with As(III). The calibration plot was established and LOD was estimated using a linear regression analysis using the formula:

$$ LOD = \frac{3 \alpha }{b}, $$

where *α* is the standard deviation of the biosensing interaction, which was measured from the calibration curve y-residuals, and ‘*b*’ is the biosensor sensitivity, which was calculated from the calibration curve's slope^46^.

Additionally, to control for field implementation, we performed an interactive analysis with an extract from the spiked rice plant on the aptamer-functionalized sensor. The extract was prepared by mixing 10 µl of extract from mature rice grains with 90 µl of PBS (0.1 M). The sample solutions were analyzed according to the analytical procedure and the recovery was calculated with the following equation:

$$ \left[ {{\text{recovery}}\, \, \left( \% \right) \, = \, \left( {{\text{spike}}\,{\text{ result }} - {\text{ raw}}\,{\text{ result}}} \right)/{\text{spike }}\,{\text{added }} \times { 1}00\% } \right]. $$

Then, we defined the stability of the aptasensor by measuring the I–V conductance once a week, in both silica nanoparticles and graphene modified Al IDEs for four consecutive weeks. This ensured the reproducibility and precision of the developed aptasensor.

## Results

### Characteristics of silica nanoparticles and graphene

#### FESEM, FETEM and XRD analysis

**Figure 1:**
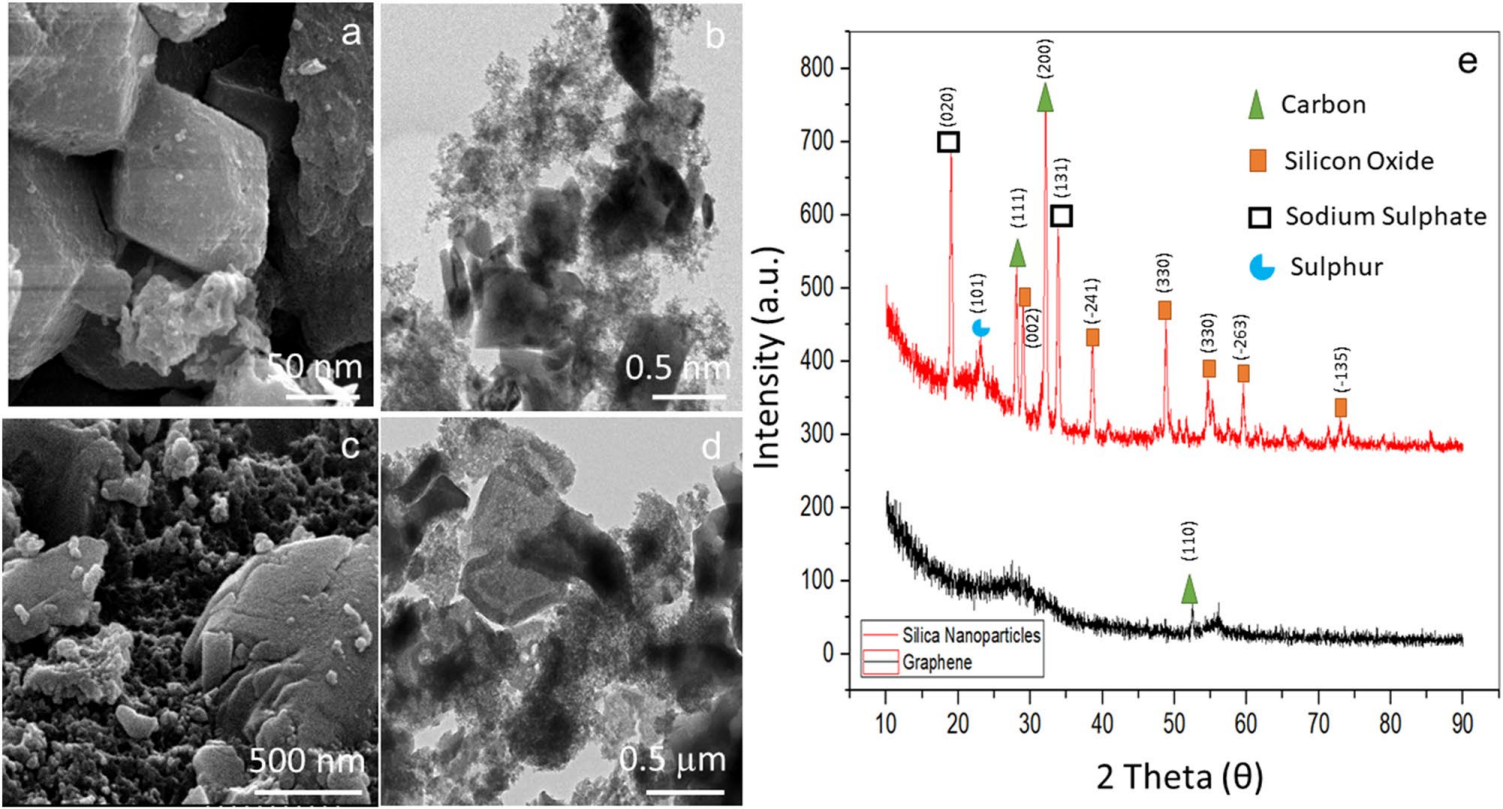
Morphological characterization using high resolution microscopy. Images captured for silica nanoparticles with FESEM (**a**) & FETEM (**b**); Graphene with FESEM (**c**) & FETEM (**d**). X-ray diffraction spectra (**e**). The comparison of diffraction peaks received between silica nanoparticles and graphene is displayed.

All identified morphologies were analyzed using a sophisticated type of high-resolution images. As shown in Fig. 1a, silica nanoparticles were observed with clearly images with irregular shape. Based on images received, the measurement of particle size was calculated at the range at 50–100 nm. Followed with FETEM image for silica nanoparticles was also observed (Fig. 1b). It can conclude that silica nanoparticle surface is porous and dense. Additionally, different silica sizes and shapes are contributed to the incomplete core reaction as a result of varying age and temperature^46,47^. Apart from that, nano-sizing of silica nanoparticles was obtained after undergoes with centrifugation processes and nano-filtration procedures^48^. Next, the comprehensive morphology analysis was continued to examine the graphene. Based on FESEM analysis images as shown in Fig. 1c, the formation of graphene was formed as flakes with average diameter size up to 2000 nm. With high resolution at nanoscale analysis, FETEM presented rough and porous nanostructure images as shown in Fig. 1d. The captured images explained that the graphene is with rough and porous structure. The appeared structures might be due to the application of KOH that was mixed with rice straw ash^49^. Along with, heating at a high temperature in a muffle furnace results in a material with a greater surface area and a higher mesoporosity^50^.

XRD patterns of synthesized silica nanoparticles have shown the presence of additional elements, including silicon oxide (JCPDS: 01-075-1381), sodium sulfate (JCPDS: 01-083-1570), sulfur (JCPDS: 01-076-2242), and carbon (JCPDS: 01-076-2242) (JCPDS: 00-018-0311) as shown on Fig. 1e. According to this pattern, silicon oxide is the most prominent element compared to other elements with large peaks at 29°, 32°, 38°, 50° 55° 60° and 74°. The sodium sulfate element was discovered to be the second most abundant, followed by sulfur and carbon. Based on the availability of the compound, stoichiometry reaction demonstrated is acceptable, where silicon Oxide is a byproduct of the reaction, and the amorphous pattern of sodium sulfate has been disclosed. For graphene, diffraction peaks were portrayed around 28.57 θ, and are called amorphous carbon material (110) with JCPDS: 01-089-8496. The peaks produced are after the KOH activation of rice straw ash at 700 °C. The structural compound of the derived graphene and micropores were created in the activation process^51^. The wide peak implies that generated graph was connected by multiple non-repeat peaks of the graphic structure^52^.

### The morphological characteristics of Al IDEs’ surface

#### SEM images and EDX analysis

**Figure 2:**
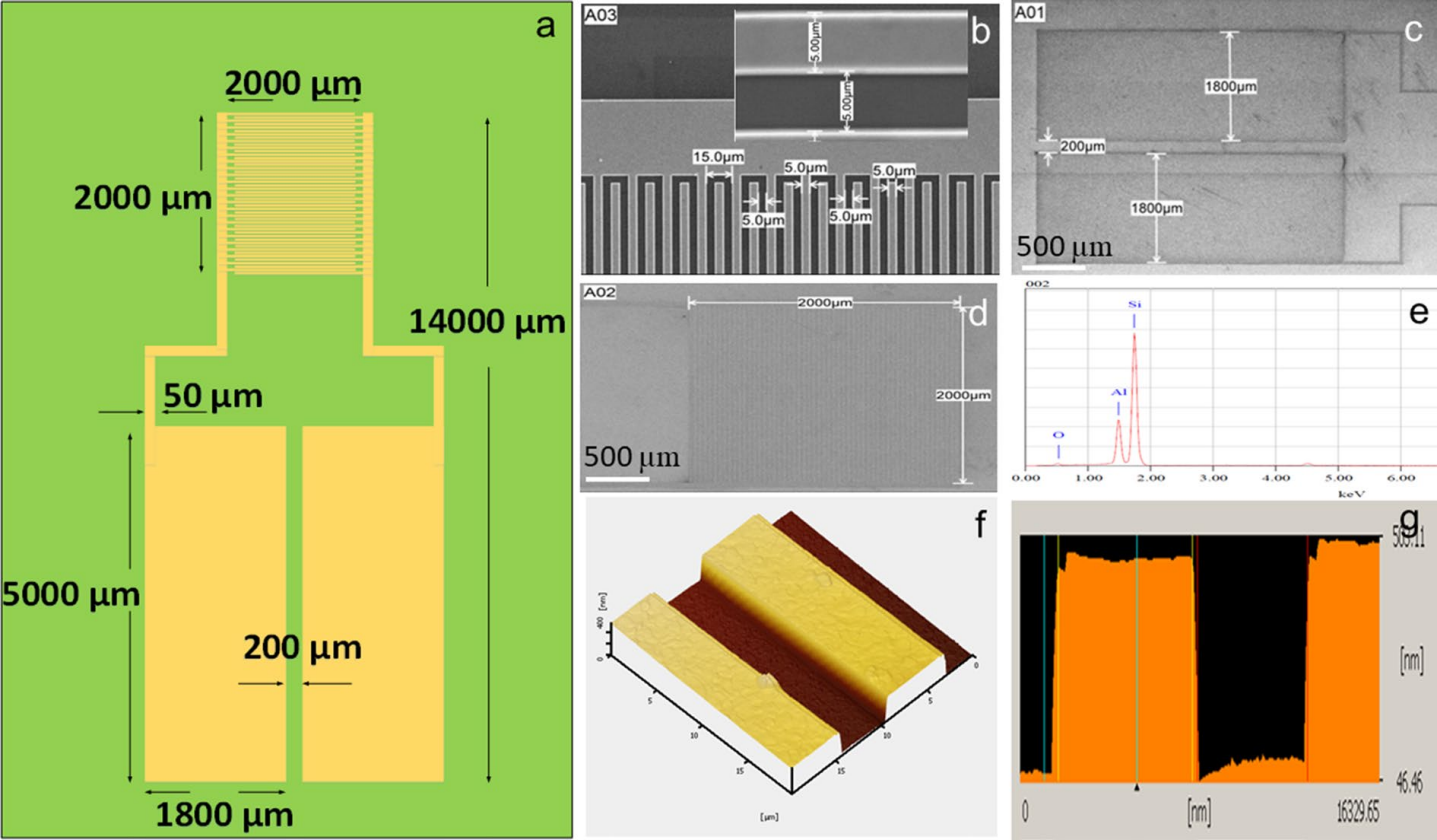
Design and characterization of bare Al IDE. (**a**) Design specifications of the proposed Al IDE were drawn using AutoCAD software. All measurements and dimensions were well presented. (**b**–**d**) Images of Al IDE under a scanning electron microscope at 5.50 and 500 µM. The fingers of channel gaps, the electrode pad, and the working electrode of the Al IDE are presented. The result indicated that the fabricated electrode followed all the specifications and dimensions provided by the layout scale. The figure inset represents a magnification of an SEM image. (**e**) EDX pattern analysis indicated the device contained the elements Si, Al, and O. (**f**) AFM topography images showed in detail that the finger gaps did not suffer any damage. (**g**) The height of the finger gap was measured starting at the bottom line to confirm the specifications of Al IDE.

In this analysis, the morphological characterization of the finger gap pattern with the electrodes was conducted on bare Al IDEs observed based on design specifications drawn using AutoCAD software (Fig. 2a). The Al IDEs were visualized around the gap between the fingers, which is 5.0 µm long (Fig. 2b), while the electrode pad measurement was visualized widthwise (1800 µm; Fig. 2c). The sensor surface was 2000 × 2000 µm^2^ (Fig. 2d). SEM revealed that the fabrication of AI IDEs in terms of gap sizes and finger/electrode length was precise at a micrometer scale, resulting in a clear shape and pattern, as the predicted result generated and designed on AutoCAD software.

Then, the EDX analysis was applied to screen the material on bare Al IDE. Based on the selected point (002) inset the (Fig. 2e), three main elements were spotted: silicon (Si) with the highest expression, followed by aluminum (Al), and, finally, oxygen (O). Since Si is the substrate base material for the Al IDE it was expected to have high expression; Al, second in expression, was deposited on the oxidized silicon (SiO_2_) surface during fabrication.

#### Atomic force microscopy

With AFM, the sensor surface of bare Al IDEs was recorded and analyzed using the tapping mode. As shown in Fig. 2f, the electrode was recorded from both top and side views, which permitted the selection and analysis of several points to determine the height of finger gaps. In Fig. 2g, it can be seen that we determined the height of the finger gap by measuring from the bottom (blue line) with $\pm$ 400 nm height and matching with the purposed measurement. Then measuring from the red line we validated that the distance of the gap was 5000 nm, coinciding with the predicted value. Lastly, starting measuring from the yellow line, we found out that the difference in the average height of bare Al IDEs is $\pm$ 10 nm.

### Electrical characterization of Al IDEs

#### I–V characterization of bare Al IDEs

**Figure 3:**
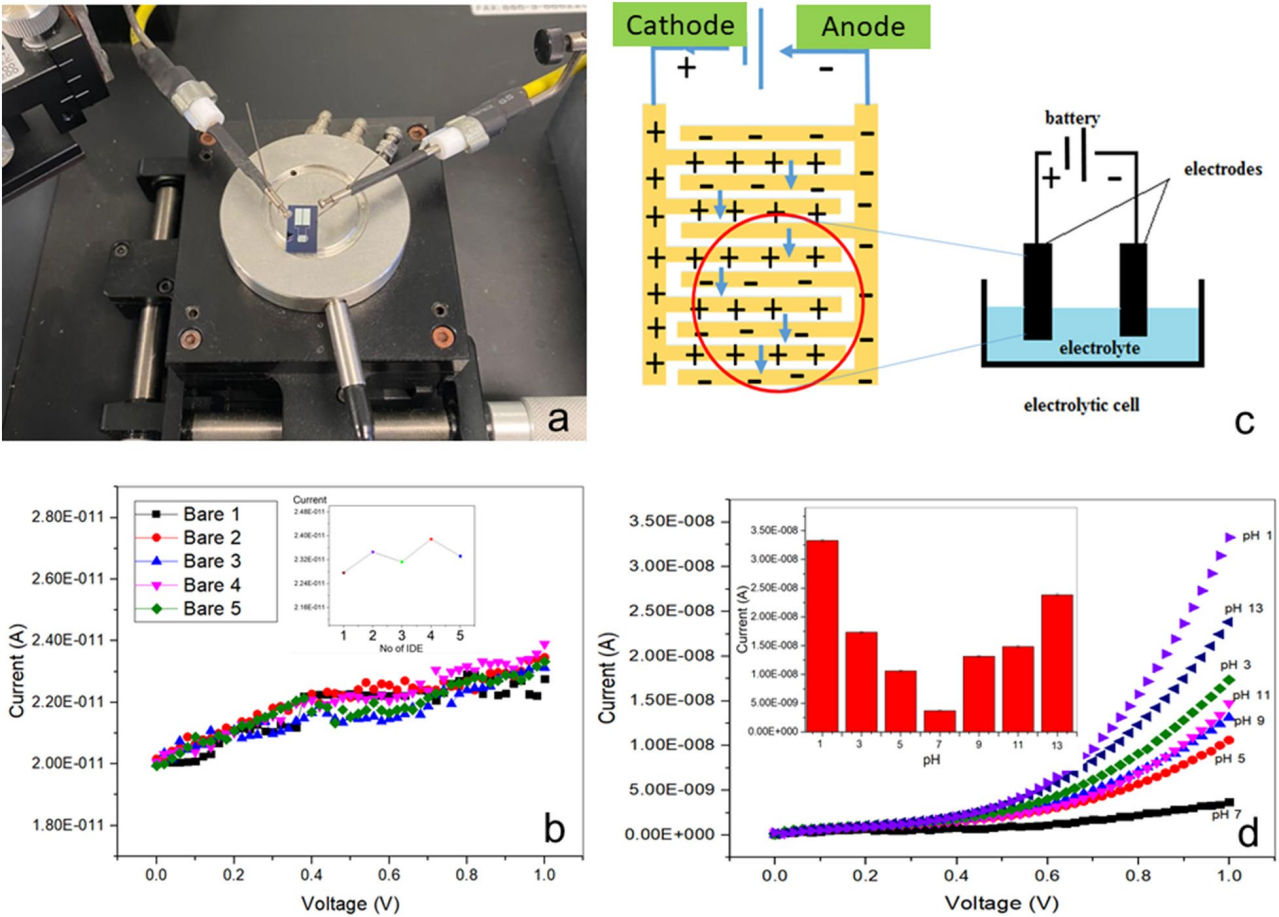
Electrical characterization measurements with a Source Meter Keithley 2450, using the Kick Start software. (**a**) Probe station with Al IDE biosensor. (**b**) I–V characteristics of bare Al IDE with Si substrate. The figure inset indicates the reproducibility using five bare Al IDEs. (**c**) Electrolysis is a procedure where an electric current is passed through a liquid to produce a chemical transition. The experiment was performed in an electrolytic system, an apparatus composed of positive and negative electrodes separated and immersed in a solution of positive and negative ions. In this case, the mechanism for transferring ions between Al IDE finger electrodes. (**d**) I–V measurements with different pH ionic solutions. The electrodes were tested in environments ranging from acidic (pH 1, 3, 5) to alkaline (pH 9, 11, 12). The figure inset indicates the difference in the current corresponding to each ionic solution and its respective pH.

In this experiment, five bare AI IDEs were tested for the electrical characterization of the sensor surface of Al IDEs. As shown in Fig. 3a, a picoammeter voltage source (0–1 V) was used to test if the voltage supply was higher than the suitable for measurement, which can cause damage to the sensor surface. A drastic change in the current variation was recorded along the analysis. The graph for the five bare Al IDEs shows a similar pattern of results throughout the voltage range, with a minor current changed difference at 1 V (Fig. 3b). The currents applied on the electrodes corresponded to 22.8, 23.5, 23.1, 23.9, and 23.3 pA (see inset graph of Fig. 3b). The difference in current from lowest to highest was 0.05 A and the average current recorded was 2.33 A. Therefore, we confirmed that the Al IDEs were appropriately fabricated without any fault or leak. Apart from that, the current variation between individual AI IDEs was only slight, confirming that the sensor surface had reproducible functional characteristics.

#### I–V characterization by electrolyte scouting

Next, the sensor surface of bare AI IDEs was analyzed for electrolyte fouling/non-fouling, using buffer solutions of different pH levels. Three acidic (pH 1, 3, and 5), one neutral (pH 7), and three alkaline (9, 11, and 13) pH levels were applied. I–V measurements started from the most acidic to the most alkaline solutions successively. Previous research has shown that the different levels of pH contain different ranges of ions, thus, leading to variable electrolyte abundance and a consequent change in conductivity^53^. The mechanism used in this pH evaluation was based on ion transfer across the sensor surface of the AI IDEs. First, we injected electric current through a chemical compound containing positively and negatively charged ions, with the droplet covering the entire surface corresponding to the finger electrodes of the Al IDE. Because of the electric field, the ions pass through the solution by generating potential differences on the electrode. Figure 3c shows how the negatively charged ions migrate towards the anode, while the positively charged ions travel towards the cathode. Furthermore, as neutral atoms and electrons move through the circuit, the neutralization and isolation of ions occur at the electrode's surface^54^. In this vein, solutions of varied pH values were utilized to determine the reaction of the electrodes in the non-fouling sensing potentials^55^. The response for each pH level was different (Fig. 3d). More specifically, we observed that pH 7 (3.71 E^−9^ A) received a low current response as compared to the other pH levels. When the pH scale values changed from low to high levels in both acidic and alkaline conditions (i.e., the most acidic or the most alkaline values), the current values also increased drastically; for instance, in the acidic trials, pH 5 corresponded to low values of current (1.06 E^−8^ A), slightly increasing when at pH 3 (1.73 E^−8^ A), and, finally, having a sharp increase at pH 1 (3.33 E^−8^ A). The same applies for the alkaline trials: a slow current increase from pH 9 (1.31 E^−8^ A) to pH 11 (1.48 E^−8^ A) resulted in a sharp increase at pH 13 (2.38 E^−8^ A). Based on these values, both extreme acidic and alkaline values increased considerably the conductivity of Al IDEs. This phenomenon is due to the high availability of H+ ions at pH 1 compared to pH 3 and pH 5. When H+ ions increase then the positive charge increases as well, thus leading to an increase in the current flow^56^. Similarly, for the alkaline scale, the availability of OH+ is higher at pH 13 as compared to the other two conditions tested here. When the concentration of OH− is high, the corresponding negative charge is increased and current flow is generated^57^.

### Detection of arsenic with Al IDEs

#### Surface functionalization and aptamer immobilization

**Figure 4:**
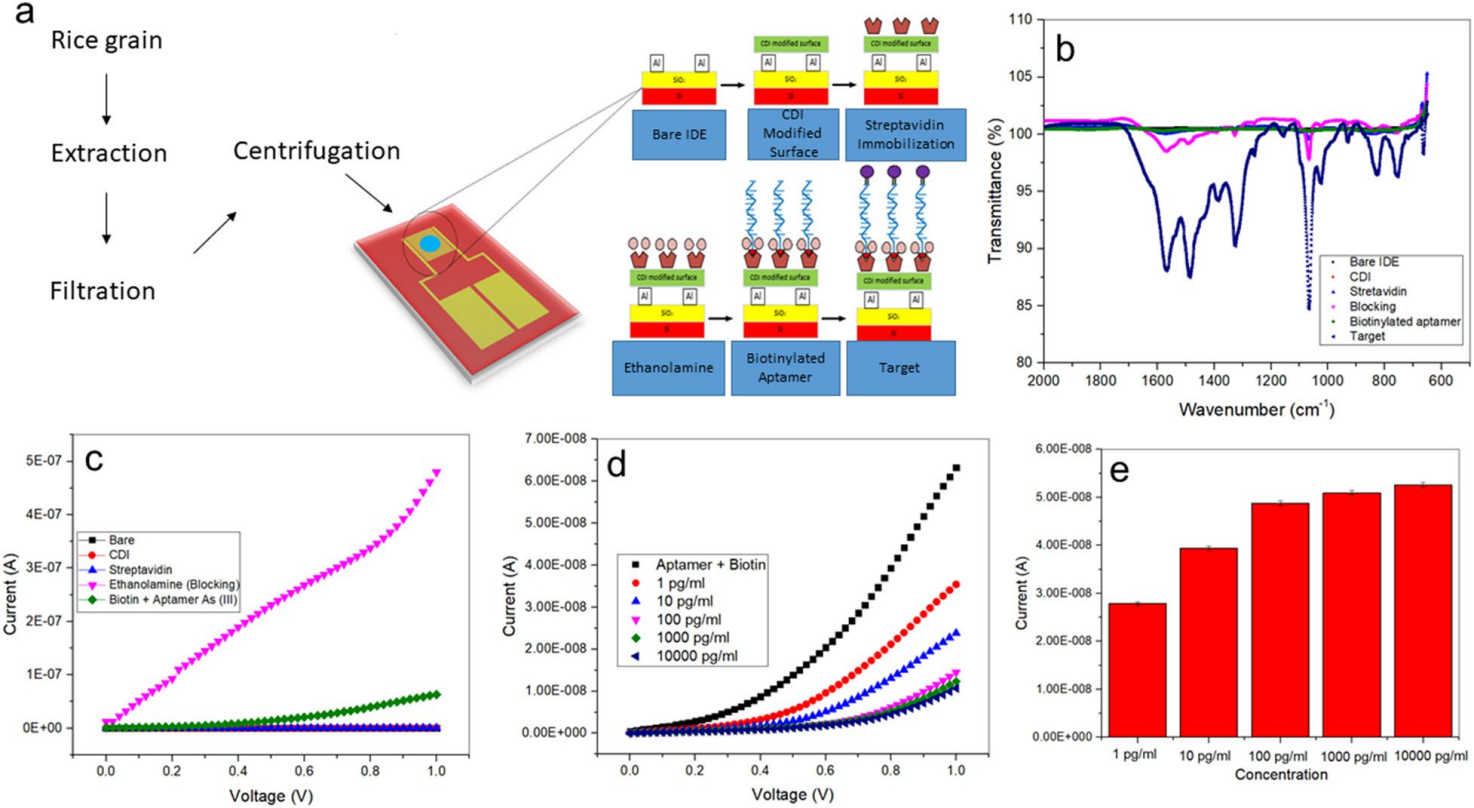
Schematic representation of the strategy for the detection of arsenic using Al IDE. (**a**) Sample preparation of rice grains. At first, 1.3 g of grains were smashed into small pieces and placed in conical flasks. Next, the samples were perfused with 15 ml of nitric acid, followed by 5 ml perchloric acid, and heated at 90 °C. At this stage, the samples acquired a yellow color and they were heated and filtered. All filtered solutions were diluted with 50 ml of distilled water and ready to be used. (**b**) I–V measurement based on surface immobilization and functionalization. CDI and streptavidin were used to modify the sensor surface of Al IDEs. Then, the remaining surface was blocked by ethanolamine. The biotinylated aptamer was immobilized on the IDE sensing surface. (**c**) FTIR analysis was used to evaluate the surface functionality at every stage of the modifying process of the Al IDE until reaching the target measurement. (**d**) Analysis of the interaction between biotinylated aptamer and arsenic at concentrations ranging from 0.01 µg/ml to 0.001 ng/ml. (**e**) Current measurements at the different concentrations of arsenic using the biotinylated aptamer Al IDEs.

Before detecting arsenic, mature rice grains were prepared with sample digestion as illustrated in Fig. 4a. Surface functionalization of bare Al IDEs was measured by the application of CDI/Streptavidin/Ethanolamine/Biotinylated aptamer/As on the 5 µM wide channel. First, the surface of the Al IDEs was analyzed by fourier-transform infrared spectroscopy (FTIR). In this analysis, each step on the sensor surface was analyzed thoroughly from spectra value at wavenumber obtained. Only the target (blue line) showed an obvious difference to the other lines and revealed plenty of peaks, reflecting the complex structure material on the target line (Fig. 4b). The early bands were recorded at 1567, 1516, and 1488 cm^−1.^ All these bands represent the double bond region which contains a carbonyl group^58^. The bands peaks at 1406, 1384, and 1360 cm^−1^ represent the OH bond, the methyl (CH_3_), and the common inorganic ions of nitrate^59^. The first sharp band at 1324 cm^−1^ represents the secondary aromatic amine with C–N stretching, while the second peak exhibits a strong and sharp bend at 1065 cm^−1^, representing the silicon–oxy compounds (Si–O–C)^60^. The last band peaks was located at 1022, 827, and 752 cm^−1^ for methylene (> CH_2_) as saturated aliphatic in form of cyclohexane ring vibrations and the last two from an aromatic ring, which specifically as C–H 1,4 and C–H 1,2 from aromatic ring (aryl)^58^.

The I–V measurement, where each reagent was analyzed separately, showed that the lowest current was recorded from the bare Al IDE (2.15 E^−11^ A) at control conditions; this was followed by streptavidin (5.05 E^−10^ A), CDI (6.08 E^−10^ A), biotin-aptamer (6.32 E^−8^ A), and the highest current was recorded from the ethanolamine condition (6.32 E^−8^ A) (Fig. 4c). These results imply that ethanolamine acts as a blocking agent as shown by the high current compared to the biotinylated aptamer and the other compounds tested here. The high current value obtained from blocking reagents is due to the high content of the hydrophilic coating, which makes it effective as a blocking agent^61^.

#### I–V measurements on the biotinylated aptamer interacting with arsenic

The detection and performance of the biotinylated aptamer were measured by the I–V changes when interacting with arsenic at various concentrations, followed by the determination of sensitivity and limit of detection (LOD). First, the I–V was measured without adding nanomaterial onto the biotinylated aptamer immobilized surface. Since 10,000,000 pg/ml of arsenic was detected, we applied different concentrations of arsenic from 1000 to 10,000,000 pg/ml. As shown in Fig. 4d, the increasing amount of arsenic provoked a concomitant gradual decrease in the current. The black line represents the biotinylated aptamer (6.32 E^−08^ A). After the addition of the minimum concentration of arsenic (1000 pg/ml), the current level was reduced (3.54 E^−08^ A), represented by the red line. This proves that the arsenic was bound to the aptamer. In Fig. 4e, the differences in the current measurements for each concentration of arsenic are presented. It was observed that the effect of the arsenic was different at each concentration. However, when starting from 100,000 to 10,000,000 pg/ml the differences in the current are relatively small, indicating that they approach saturation levels.

#### Enhancing detection of arsenic by AL IDE: applying a sensor surface modified with silica nanoparticles/grapheme

**Figure 5:**
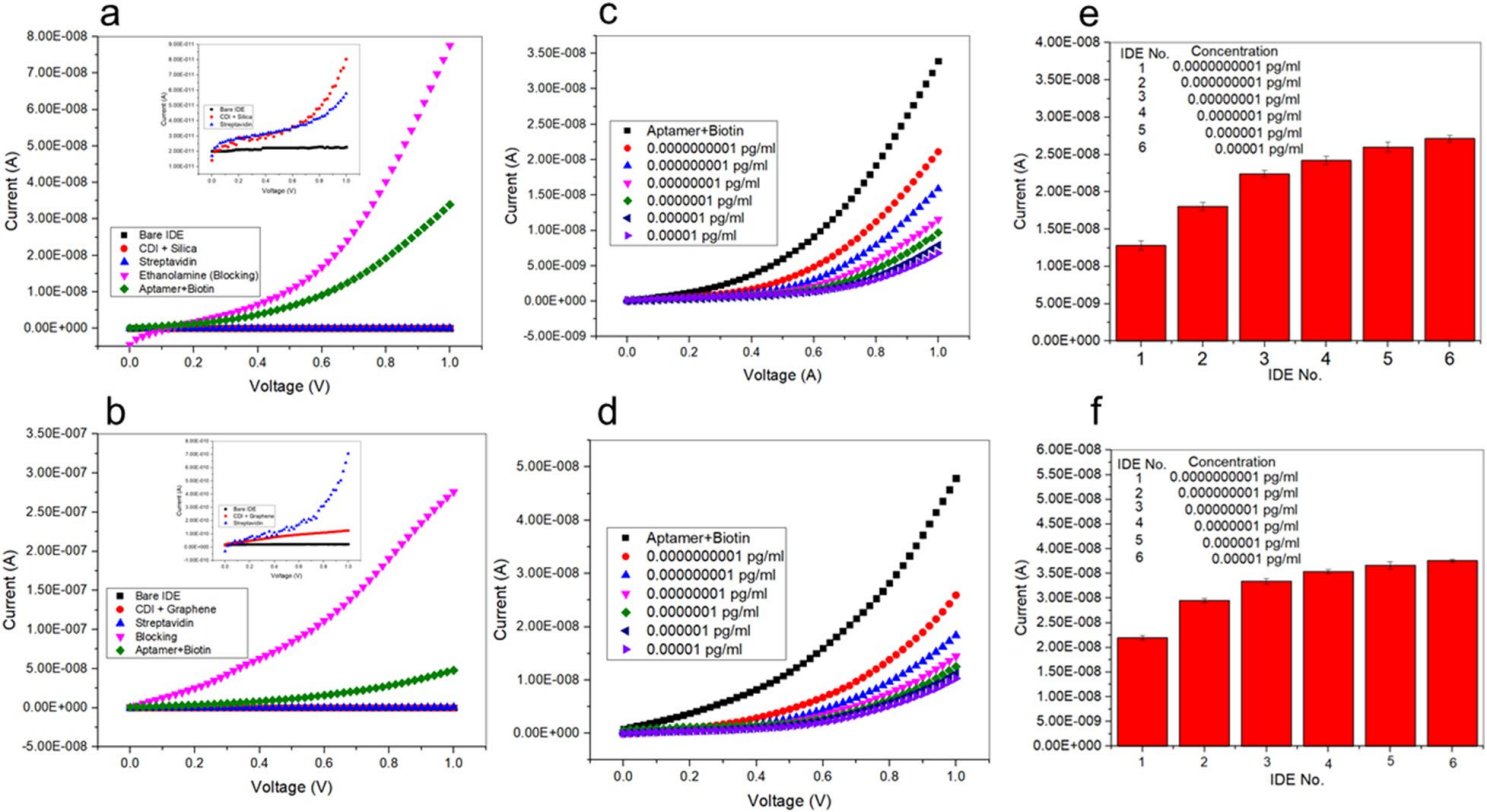
Surface functionalization measured by voltammetry signal. The inset graph represents the current values recorded with bare, CDI-silica or CDI-graphene, and streptavidin Al IDEs modified with (**a**) silica nanoparticles and (**b**) graphene. The figure insets represent enlarged portions of bare IDE, CDI-Silica or CDI-Graphene and Streptavidin. We evaluated the impact of the interaction between (**c**) CDI-silica nanoparticles or (**d**) CDI-graphene. The current measurements for different concentrations of arsenic detected with (**e**) CDI-silica and (**f**) CDI-graphene Al IDEs are presented here.

Initially, we evaluated the electrical potential of the CDI-silica nanoparticles/graphene conjugates and other molecular immobilizations on the Al IDE surface. Both bare Al IDE had the lowest current level (2.27 E^−11^ A). When CDI-silica or CDI-graphene were attached independently onto the surface of IDE, the latter displayed higher electrical conductance (7.07 E^−10^ A) than the former (8.03 E^−11^ A) (Fig. 5a,b). The difference in the conductance of the two nanostructures was small, emphasizing their potential as materials presenting high electrical conductivity. When using immobilized streptavidin, the current recorded for graphene (1.25 E^−10^ A) and silica nanoparticles (5.78 E^−11^ A) showed that they continued conducting electricity at satisfactory levels. The remaining free surface was masked with ethanolamine to avoid non-specific arsenic binding. With the ethanolamine on the sensor surface, the current was drastically increased for both graphene (2.76 E^−7^ A) and silica nanoparticles (7.76 E^−8^ A). The current recorded under ethanolamine and aptamer-biotin-streptavidin conditions for silica nanoparticles and graphene were 7.75 E^−8^ and 2.75 E^−7^ A, respectively. These differences are due to variations in the size and shape of silica nanoparticles and graphene. Upon use of the biotin–aptamer complex, the electrical intensity was decreased because of the charge induced by the complex itself. This attachment onto the sensing surface was considered to be a complete sensing area to detect arsenic.

Nanotechnological materials play a significant role in the retention of nanoparticles, like silica and graphene^62^. Characterization studies of the surface charge of nanoparticles by measuring their zeta potential and their sizes on the stability of nanoparticles at solid interfaces have been conducted, especially in the context of sensor development^36,37^. In the case of molecular absorption, the interactions play an important role, since the larger the particles the more intense the interface^63^. For example, graphene here had a size of up to 2000 nm, which covered largely the available surface and presented high porosity. Additionally, graphene has the potential to achieve high selectivity through molecular size exclusion effects, due to its high permeability at a low thickness, increasing the practicability of graphene-based sensors^64^. To further enhance the utility of silica nanoparticles, surface modification and immobilization techniques that help the pairing of silica nanoparticles with a variety of biomolecule targets can be applied. For instance, the silica nanoparticles can bind to probe biomolecules, such as oligonucleotides, enzymes, antibodies, or other proteins; all these have been used for bioanalytical detection^65^. A distinct benefit of the silica matrix/surface is its adaptability to various surface modulation protocols: avidin, sulfide, amine, or carboxylate groups can be added to the silica surface. Avidin may be passively adsorbed by the silica surface and, then, cross-linked with glutaraldehyde. Due to the close affinity between avidin and biotin, the resulting avidin-coating of nanoparticles could be conjugated with biotinylated molecules. Due to silica's affinity for avidin, the silica can be used in assays of regularly used and readily accessible biotinylated compounds^66^.

Next, to evaluate the interaction between CDI-silica nanoparticles/graphene, serial dilutions of arsenic from the atto- to the femtomolar level were applied on the modified sensor surface. As displayed in Fig. 5c,d, both Al IDEs modified with nanomaterials presented an identical response pattern to the ascending arsenic concentrations: the current intensity declined due to the binding of arsenic to the aptamer. However, each concentration caused different values current for each of the modified sensor surfaces. The highest current change was noted with the graphene modification as compared to silica nanoparticles, which can be attributed to the distinctive electron transport and high adsorption properties of graphene (Fig. 5e,f). In this case, As(III) ions were adsorbed by the surface of the electrode and reduced to As(0) by the employment of the applied deposition potential. This deposition process and the conductivity can be attributed to the active groups like the biotin conjugated with aptamer for both modifications.

#### High‑analytical performance of silica nanoparticles/graphene modified aptasensor at the detection of arsenic

**Figure 6:**
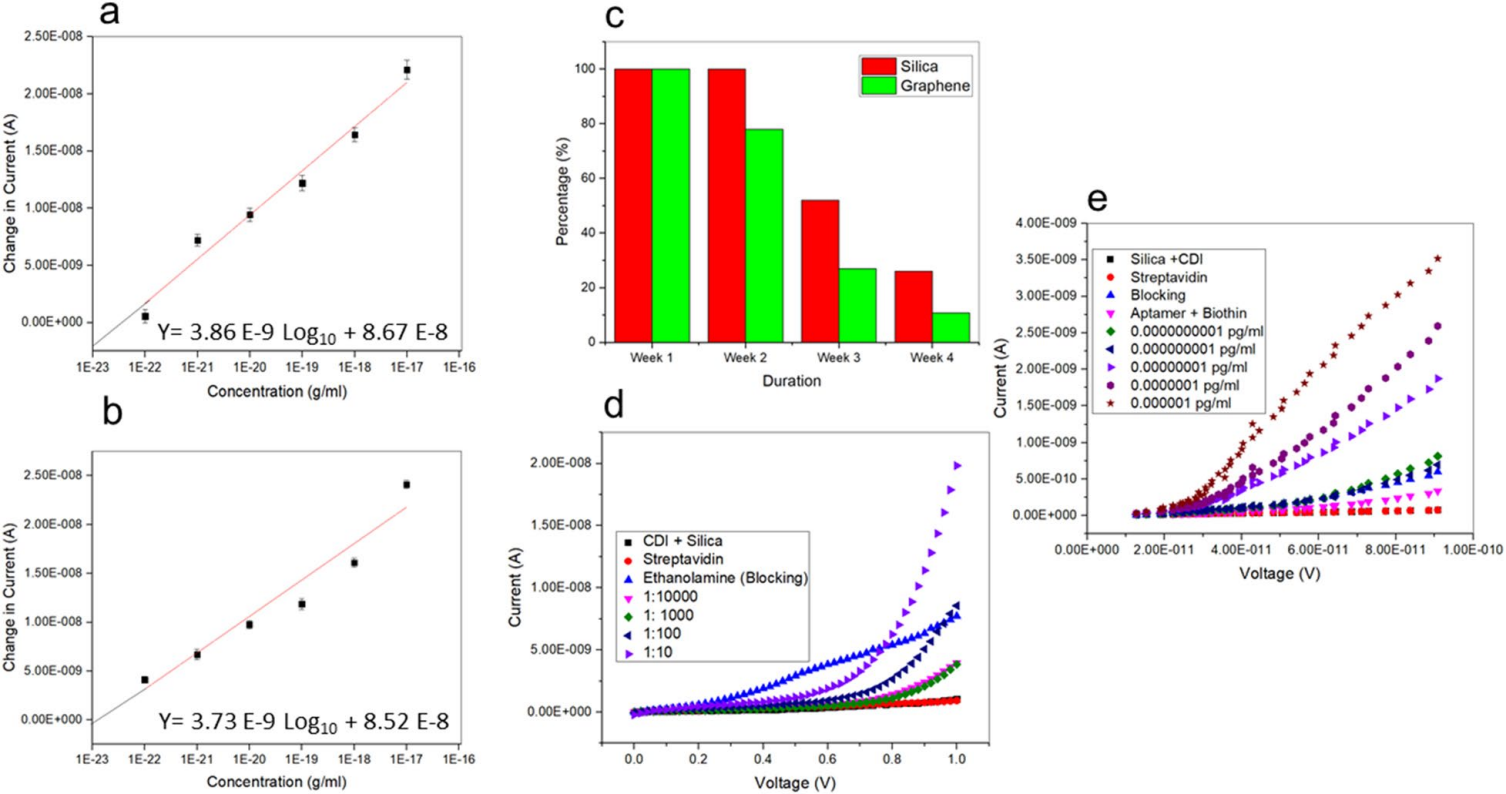
The current changes at various arsenic concentrations at 1 V, indicating the limit of detection (LOD) measured after modifications with (**a**) silica nanoparticles or (**b**) graphene. Analysis of spikes of the mature rice grains was conducted to determine the realistic application of silica or graphene nanomaterials-modified sensor surface for the detection of the target analyte, As(III). (**c**) A stability test was conducted for four weeks using I–V measurement to evaluate the reproducibility and precision of the developed sensor for both nanomaterials. (**d**) Determination of non-fouling in the extract of rice grains at a dilution of 1:100. (**e**) Analysis of spikes with a real sample using the silica nanoparticles modification.

In this study, a linear calibration curve was generated to analyze the limit of detection (LOD) and the sensitivity level of the generated Al IDE aptasensor. The LOD is used to quantify and readily detect the lowest concentration of the analyte given, whereas the sensitivity is defined as the slope of the calibration curve^67^. Apart from that, the performance of detection with silica nanoparticles/graphene and the calibration curve were evaluated. The different arsenic concentrations applied in these experiments varied from 0.0001 fg/l to 0.01 pg/l. Subsequently, the calibration curve equation was ΔI (A) = 3.86 E^−09^ log (Arsenic concentration) [g/ml] + 8.67 E^−08^ [A] for silica nanoparticles, whereas for graphene was described by Y = 3.73 E^−09^ (Arsenic concentration) [g/ml] + 8.52 E^−08^ (Fig. 6a,b). The performance of both modifications exhibited a high sensitivity as described by the determination coefficient (R^2^), which equaled 0.98 for the silica nanoparticles and 0.94 for the graphene. These results, being close to the maximum R^2^ (1), indicate that the precision of the measurement is better in the case of silica than in the graphene nanoparticles^68^. Apart from that, the LOD was predicted for silica nanoparticles and for graphene 0.0000001 pg/ml, when calculated using 3σ/slope and indicated as the y-intercept.

The stability and the accuracy of the developed sensor for each of the two nanomaterials were evaluated once a week for four consecutive weeks by I–V measurement and using a single Al IDE as shown in Fig. 6c. The changes occurred for both nanomaterials during week 2, where graphene´s 100% dropped to 78% compared to the initial measurement, whereas silica-modified Al IDE still performed at 100%. During weeks 3 and 4, the stability of both nanomaterials dropped for silica nanoparticles from 75 to 26% and for graphene from 47 to 11%. From this analysis, we can conclude that AI IDE modified with silica nanoparticles preserved the stability for a longer period as compared to the graphene modification.

#### Performance of the sensor in the analysis of a real sample

Based on the longer stability and the smaller size of silica compared to graphene, properties that make easier its use in soil, as well as its spherical shape compared to the flakes´ shape of graphene, we decided to further test the performance of silica modification on real samples. In this experiment, we diluted the rice grain samples (1.3 g/ml) at concentrations ranging from 1:10 to 1:10,000, and the changes in current were monitored (Fig. 6d). Increasing dilutions provoked a concomitant decrease in the current from 1.98 E^−8^ (1:10), 8.56 E^−9^ (1:100), to 3.84 E^−9^ (1:1000), and, finally, to 3.02 E^−9^ A (1:10,000). The 1:10 dilution revealed notable biofouling, with the 1:100 dilution showing a higher non-fouling.

These results show that the Al IDEs can detect arsenic in a real sample of rice grains. The result is portrayed by the presence of interference by other major element abundance in the spiking sample. In recent years, the application of nanomaterials in various fields of research and technology has attracted more attention, due to the multitude of their capabilities. So, here, we decided to examine the potential of silica nanoparticles in the analysis of spikes in a 1:100 diluted sample. As shown in the present study, silica nanoparticles proved to be effective in detecting arsenic^69^, as measured by the changes in the current intensity. This activity can be attributed to the existence of a moiety that strongly reacted with the positively charged target analyte^70^. This means that the peak reaction is determined by the amount of As(III) ions adsorbed by the modified sensor surface of the electrode, and silica nanoparticles can also be applied to the analysis of spikes.

#### Recovery analysis with an extract from rice grains

As shown in Fig. 6e, the analysis of spikes using a real sample demonstrated high values of affinity as compared to samples with no spikes. At a concentration of 0.00000001 pg/ml, the current presented high values (3.51 E^−9^ A) as compared to the non-spiking analysis (7.93 E^−9^ A). As concentration increased to 0.000000001 pg/ml the measured current increased. Apart from that, a calculation for the recovery rate was performed, where the functionality of the sensor in the extract presented a recovery rate of up to 120%. This is similar to other reports where a slightly higher current was measured under the analysis of spikes and recovery test when compared to the application of buffer solution^71^.

## Conclusion

This study presents a promising aptasensor for the detection of As(III). We evaluated the sensor surface with the use of silica nanoparticles/graphene by applying electrochemical techniques. The Al IDEs performed well in the physical and electrical characterization tests. As for the surface functionalization and the aptamer immobilization, FTIR results confirm the precise surface enhancement using a biotinylated aptamer specific to target arsenic. The silica and graphene nanoparticles-modified Al IDEs presented an excellent performance at the lowest detecting limit as calculated by the 3σ/slope 0.0000001 pg/ml with R^2^ almost equal to 1 for both nanomaterials. Also, the electrodes were successful at detecting As(III) in real samples, being a promising technique for environmental analyses. Further, Al IDEs modified with silica nanoparticles performed better in the analysis of spikes than graphene. Apart from that, the electrodes with modified silica nanoparticles were more stable than graphene ones, within a 4-week window. The present research provides evidence that the modified Al IDEs can serve as an efficient strategy for a rapid, accurate, highly responsive, and selective detection system for As(III) in environmental samples.
